# Overnight orthokeratology is comparable with atropine in controlling myopia

**DOI:** 10.1186/1471-2415-14-40

**Published:** 2014-03-31

**Authors:** Hui-Ju Lin, Lei Wan, Fuu-Jen Tsai, Yi-Yu Tsai, Liuh-An Chen, Alicia Lishin Tsai, Yu-Chuen Huang

**Affiliations:** 1 Department of Medical Research, China Medical University Hospital, No. 2 Yuh Der Road, Taichung 404, Taiwan; 2 School of Chinese Medicine, College of Chinese Medicine, China Medical University, Taichung, Taiwan; 3 Department of Ophthalmology, China Medical University Hospital, Taichung, Taiwan

**Keywords:** Atropine, Axial length, Cornea endothelium, Myopia, Orthokeratology

## Abstract

**Background:**

Many efforts have been invested in slowing progression of myopia. Among the methods, atropine administration and orthokeratology (OK) are most widely used. This study analyzed the efficacy of atropine and OK lens in controlling myopia progression and elongation of axial length.

**Methods:**

This retrospective study included 105 patients (210 eyes) who wore OK lenses and 105 patients (210 eyes) who applied 0.125% atropine every night during the 3 following period. Student *t*-test, linear regression analysis, repeated measure ANOVA, and Pearson’s correlation coefficient were used for statistical analysis.

**Results:**

The change in axial length per year was 0.28 ± 0.08 mm, 0.30 ± 0.09 mm, and 0.27 ± 0.10 mm in the OK lens group, and 0.38 ± 0.09 mm, 0.37 ± 0.12 mm, and 0.36 ± 0.08 mm in the atropine group for years 1, 2, and 3, respectively. Linear regression analysis revealed an increase in myopia of 0.28 D and 0.34 D per year, and an increase in axial length of 0.28 mm and 0.37 mm per year in the OK lens and atropine groups, respectively. Repeated measure ANOVA showed significant differences in myopia (*p* = 0.001) and axial length (*p* < 0.001) between the atropine and OK lens groups; in astigmatism, there was no significant difference in these parameters (*p* = 0.320). Comparison of increases in axial length in relation to baseline myopia showed significant correlations both in the OK lens group (Pearson’s correlation coefficient, *r* = 0.259; *p* < 0.001) and atropine group (*r* = 0.169; *p* = 0.014). High myopia patients benefited more from both OK lenses and atropine than did low myopia patients. The correlation of baseline myopia and myopia progression was stronger in the OK lens group then in the atropine group.

**Conclusions:**

OK lens is a useful method for controlling myopia progression even in high myopia patients.

## Background

Myopia is the one of the most common ocular disorders in the world. The prevalence of myopia is about 20%–30% in North American, Australian, and European populations [1-3], and much higher (40%–70%) in the Asian population [4-6], especially in China [7-9]. Myopia is an important public health problem because it is associated with increased risk for chorioretinal degeneration, retinal detachment, and other vision-threatening abnormalities [10,11]. Several therapeutic methods exist for the correction of myopia, such as corrective spectacles, contact lenses, keratorefractive surgeries, intraocular lenses (IOLs), and clear lens extraction [12-14]. However, possible sequel of keratorefractive surgeries of high refractive errors, such as glare, halo, and contrast sensitivity have been reported [15,16]. Therefore, prevention of high myopia is of utmost importance.

The nonselective muscarinic acetylcholine receptor (mAChR) antagonist, atropine, slows down myopia progression in a dose-dependent manner as compared with that in a placebo-treated group [17-19]. Atropine has proved useful both in animal studies and human clinical trials and is now widely used to control progression of myopia. However, myopia is never completely resolved. In atropine users, increasing intraocular pressure and photo-stress of the crystalline lens and retina are often concerned; photophobia and poor near vision are often to be confronted with. Moreover, rebound effect after atropine cessation had been mentioned before [20]. After stopping treatment, eyes treated with atropine demonstrated higher rates of myopia progression compared with eyes treated with placebo. However, the absolute myopia progression after 3 years was significantly lower in the atropine group compared with placebo [20].

Orthokeratology (OK) uses specially designed rigid contact lenses to reshape the cornea in order to temporarily reduce or eliminate refractive error [21,22]. Modern OK using sophisticated contact lenses with a reverse-geometry design can provide faster, larger, and more predictable refractive changes than OK lenses used in the original method introduced in the early 1960s [23]. Overnight OK lenses can decrease the patient’s need to wear contact lenses or spectacles in the daytime by providing acceptable vision for normal routine activity. The presumed mechanisms of OK lens-induced myopic reduction include central corneal flattening, thinning of the central corneal epithelium [24,25], thickening of the mid-peripheral cornea, and peripheral vision myopic shift [26]. However, several studies report that OK lenses increase higher-order aberrations of the cornea and decrease contrast sensitivity [27,28]. Despite controversies related to safety issues, OK lens use is becoming increasingly popular [21].

High myopia is one of the major causes of legal blindness, and many efforts have been invested into slowing elongation of axial length and decreasing myopia progression. In this study, two of the currently most useful methods in controlling myopia are compared: OK lenses and 0.125% atropine. Refractive errors, axial length, and endothelium cell count were analyzed to determine the effects of the two groups in controlling myopia progression.

## Methods

This is a retrospective cohort study for a three years period, the patients using atropine or OK lens were grouped according to the selection of the patients themselves; no special recommendations were done in our department. The benefits and possible defects of the two methods were all informed to the patients and their families. Including photophobia, poor near vision and the risk of increasing intraocular pressure (IOP) might confront when using atropine; the risks of unstable vision in the daytime, glare at night and risks of keratitis in OK lens user. At the time of treatment, the patients and their families understood the different methods for treating myopia and selected the method themselves. Patients with complete clinical data during the study period (3 years, from March 2009 to March 2012) and undergone full and regular examinations were included in this study. All participants had a visual acuity with distance correction of 0.1 logMAR (20/25) or better. Landolt C ETDRS Distance Chart was used. The UCVA (uncorrected visual acuity) and BCVA (best corrected visual acuity) were all measured between 2 and 4 pm for each patient. We used alpha of 0.05, power of 80%, and the sample size estimated was approximately 105 subjects for each group, the patients with former ID number were included. We selected the first 210 patients (105 atropine and 105 OK lens) met the inclusion criteria and who visited our department had smaller ID number in our hospital; a total of 105 patients who used OK lens and 105 patients who used 0.125% atropine (Wu-Fu pharmaceutical Cc., Inc., YiLan, Taiwan) every night before sleep. Previous study of Wu et al. had proved that low concentration atropine is effective in controlling the progression of myopia [29], 0.125% atropine was selected in the control group because that 0.125% atropine is the lowest concentration of atropine available and marketed in our country now. Their ages ranged from 7 to 17 years and myopia ranged from 1.5 to 7.5 D. Patients received 0.125% atropine and did not discontinue the drugs for more then 10 days during the study period (3 years). The study was approved by the ethics committee of China Medical University Hospital (Taichung, Taiwan) and was performed in accordance with the tenets of the Declaration of Helsinki for research involving human subjects. Informed consent was obtained from all participants. Comprehensive ophthalmic examinations were performed before treatment and at every visit. None of the participants had ocular insult or disease such as retinopathy, prematurity, neonatal problems, a history of genetic disease, and connective tissue disorders associated with myopia such as Strickler or Marfan syndromes. Clinical examinations included visual acuity, refraction error, slit lamp examination, ocular movements, intraocular pressure, and fundoscopy. Patients with organic eye disease, a history or evidence of intraocular surgery, and history of cataract were excluded from this study (Table 1). Non-cycloplegic subjective vision and cycloplegic objective refraction recorded at the visits before commencement of 0.125% atropine or OK lens treatment (baseline) and 1, 2, and 3 years after were compared. Myopic diopter and axial length were also checked every year after discontinuing use of OK lenses for 3 weeks in the summer vocation between the semesters. The refractive error (in diopters [D]) of each individual was measured after administering one drop of cycloplegic drug (1% mydriacyl; Alcon, Berlin, Germany). The data of the patients were detected each eye and the averages of the two eyes were used for analyzing; the differences of myopia degree between the two eyes over then 2 D and astigmatism over then 1.5 D were also excluded from this study. Individuals with myopia from 1.5 to 7.5 D (average, 4.25 ± 1.5 D) and astigmatism from 0 to 2.75 D (average, 0.75 ± 0.75 D) (negative cylinder was used in this study) were included in this study; cases of extreme high myopia (over 7.5 D) and astigmatism (over 2.75 D) were excluded. Auto-refraction (Autorefractor/auto-keratometer [ARK 700A; Nikon, Tokyo, Japan]) was conducted for both eyes by experienced optometrists who were trained and certified in the study protocols. Refractive data, sphere (s), negative cylinder (c), and axis measurements were analyzed.

**Table 1:** Inclusion and exclusion criteria

**Exclusion criteria**	**Inclusion criteria**
Retinopathy	Aged: 7-18 year-old (average 10±2.3 year-old)
Prematurity	Myopia: 1.5 D to 7.5 D (average 4.25 D ± 1.5 D)
Neonatal problems	Astigmatism: 0 D to 2.75 D (average 0.75 D ± - 0.75)
History of genetic disease	Follow up: 6-40 months (24 ± 1.8 months)
Connective tissue (e.g. Strickler or Marfan syndromes)	Distance correction: 0.1 log MAR (20/25) or better
Organic eye disease	
Intraocular surgery (e.g. history of cataract)	

Patients who applied atropine ophthalmic eye drops received one drop of 0.125% atropine every night before sleep and wore glasses prescribed by a certified ophthalmologist and modified according to any refractive changes during the follow-up period. The OK lenses used in this study were 4-zone, reverse-geometry lenses (Emerald Lenses; Euclid Systems Corp., Herndon, VA, manufactured from Boston XO material; Polymer Technology Corp., Wilmington, MA) with a nominal Dk of 100 × 10^-11^ cm^2^/s) (mL O_2_/mL · mmHg). The nominal central thickness of the lenses was 0.22 mm, and the diameter was 10.4–11.0 mm. The parameters of the lenses were varied to achieve good centration and good fluorescein pattern. After the lenses were dispensed, the patients were advised to wear them every night for at least 6–8 consecutive hours. In OK lens group, the patients with myopia over then 5.75 D would use double reverse curves and dual geometric (DG) designs OK lens from Euclid Systems Corp. The patients of the 2 groups returned for examination every 3 months and underwent slit lamp examinations for any adverse events. The OK lens fit was evaluated at these visits. The first spectacles given to the patients in atropine group were when their UCVA worsen then 0.3 logMAR. OK lenses and spectacles were replaced if visual acuity was worse than 0.3 logMAR during the follow-up.

Refraction, visual acuity, axial length, and corneal endothelium cell count obtained before initiation of the treatments were used as the baseline values; measurements were monitored every 3 months thereafter and they were also checked every year after discontinuing use of OK lenses for 3 weeks in the summer vocation between the semesters. That is, refractive error in OK group was measured 3 weeks after lens cessation 3 weeks for each patient after UCVA was measured. The axial length was evaluated using a noncontact optic biometric device (IOL Master; Carl Zeiss Meditec AG, Jena, Germany). On each occasion, 5 successive measurements were obtained, and their mean was used as a representative value. The measurements data were obtained by a well-trained examiner to decrease the errors induced by different examiners. Changes in axial length were evaluated prospectively and compared. Noncontact specular microscopy of the central corneal endothelium was performed with an SP-2000 specular microscope (Topcon Co, Tokyo, Japan), and endothelial photographs were taken. Subsequently, the Topcon IMAGEnet processing system (Topcon) was used to analyze these images. The boundaries of at least 100 cells per image were digitized, and the mean endothelial cell density, coefficient of variation of cell area, and percentage of hexagonal cells were calculated by semi-automatic mode.

Data are presented as ranges or means ± standard deviation. A student *t*-test was used to compare the baseline conditions of the two groups. Linear regression, repeated measure ANOVA and Pearson’s correlation coefficient analyses were performed to compare the refractive error at baseline and increased axial length. The more significant linear correlation and higher regression coefficient (β) indicate the higher positive correlation. A *p* value <0.05 represented significant in this study.

## Results

In the OK lens group, 105 patients (53 males and 52 females) who successfully completed the 3-year follow-up examinations were enrolled. Their ages ranged from 7 to 17 years (average, 11.82 ± 1.25 years). Sixteen (15.24%) patients were aged 7–9 years, 70 (66.67%) were aged 10–13 years, and 19 (18.83%) were aged 14–17 years (Table 2). At baseline, their myopia ranged from 1.5 to 7.5 D (average, 4.25 ± 1.5 D), and astigmatism ranged from 0 to 2.75 D (average, 0.75 ± 0.75 D); logMAR uncorrected visual acuity (UCVA) was between 0.20 and 1.40 logMAR (mean, 0.80 ± 0.45), and axial length ranged from 22.05 to 27.05 mm (mean, 24.12 ± 1.25 mm; Table 2). In the atropine group, 105 patients (53 males and 52 females) used 0.125% atropine every night throughout the 3-year follow-up (Table 2). Their ages ranged from 7 to 17 years (average, 11.12 ± 1.68 years). Twenty-three (21.91%) patients were aged 7–9 years, 70 (66.67%) were aged 10–13 years, and 12 (11.41%) were aged 14–17 years. Among the 105 subjects, 90 (90.5%) patients required spectacles to perform daily activities. At baseline, their myopia ranged from 1.5 to 7.5 D (average, 4.0 ± 1.75 D; Table 2) and astigmatism was between 0 and 2.75 D (average, 0.5 ± 0.75 D; Table 2); UCVA ranged from 0.10 to 1.40 logMAR (mean, 0.81 ± 0.28; Table 2), and axial length ranged from 21.12 to 27.23 mm (mean, 24.23 ± 1.35 mm; Table 2). At baseline, the 2 groups were comparable in terms of myopia (*p* = 0.975), astigmatism (*p* = 0.897), and axial length (*p* = 0.985) (Table 2). All demographic data on UCVA, axial length, age, and gender are listed in Table 2; there were no significant differences in all the baseline conditions between the groups.

**Table 2:** Baseline data of patients in the OK lens and atropine group

	**OK**^**#**^**(mean ± SD)**	**Atropine**^**$**^**(mean ± SD)**	***p*** **value**
Age, y/o	11.82 ± 1.25	11.12 ± 1.68	0.745
Sex, M/F	1: 0.99	1: 098	0.987
Myopia (D^d^)	1.5 to 7.5 (4.25 ± 1.5)	1.5 to 7.5 (4.0 ± 1.75)	0.975
Astigmatism (D)	0 to 2.75 (0.75 ± 0.75)	0 to 2.75 (0.5D ± 0.75)	0.897
UCVA* (log MAR^b^)	0.8 ± 0.45	0.81 ± 0.28	0.982
BCVA^a^ (log MAR)	0.1 ± 0.015	0.12 ± 0.05	0.876
Axial length (mm)	24.12 ± 1.25	24.23 ± 1.35	0.985

^#^OK: Orthokeratology.

^$^0.125% atropine.

*UCVA: Uncorrected visual acuity.

^a^BCVA: Best corrected visual acuity.

^b^log MAR: logarithm of the Minimum Angle of Resolution.

^d^D: diopter.

Using linear regression analysis, we found that myopia increased by 0.28 D ± 0.18 D and 0.34 D ± 0.21 D per year in the OK lens group and the atropine group, respectively (Table 3). The change in myopia diopters per year was 0.29 ± 0.31 D, 0.27 ± 0.24 D, and 0.28 ± 0.31 D in the OK lenses group, and 0.31 ± 0.19 D, 0.35 ± 0.85 D, and 0.32 ± 0.53 D in the atropine group for years 1, 2, and 3, respectively (Table 4). The change in axial length per year was 0.28 ± 0.08 mm and 0.37 ± 0.09 mm in the OK lens group and the atropine group, respectively (Table 3). The change in axial length per year was 0.28 ± 0.08 mm, 0.30 ± 0.09 mm, and 0.27 ± 0.10 mm in the OK lens group, and 0.38 ± 0.09 mm, 0.37 ± 0.12 mm, and 0.36 ± 0.08 mm in the atropine group for years 1, 2, and 3, respectively (Table 4). There are also significant but weak differences comparing axial length and myopic degree each year and data are not showed. We also compared the changes in the group aged 10–13 years; the averaged changes of myopia were 0.29 ± 0.21 D and 0.34 ± 0.31 D in OK lens and atropine groups per years (*p =* 0.003). The averaged changes of axial length per years: OK lens and atropine groups were 0.29 ± 0.11 mm and 0.37 ± 0.12 mm, respectively (*p =* 0.0035). Astigmatism (analyzed using a negative cylinder) changed by ±0.02 D and ±0.01 D per year in the OK lens group and the atropine group, respectively (Table 3); the axis of astigmatism did not show significant changes during the study period in the 2 groups.

**Table 3:** Predictors of myopia and astigmatism between atropine and OK lens groups by linear regression analysis

**Dependent variable**		**Regression coefficient (95% confidence interval)**	**Repeated measure ANOVA (between the two groups)**
Myopia	OK lens^#^	-0.28 (-0.40 ~ -0.16)	*p* = 0.001
	Atropine^$^	-0.34 (-0.46 ~ -0.21)	
Astigmatism	OK lens^#^	± 0.02 (0.05 ~ 0.03)	
	Atropine^$^	± 0.01 (0.05 ~ 0.02)	
Axial length	Ok lens^#^	0.28 (0.20 ~ 0.36)	*p* < 0.001
	Atropine^$^	0.37 (0.29 ~ 0.44)	

^#^OK: Orthokeratology.

^$^0.125% atropine.

**Table 4:** Increase of myopia, stigmatism and axial length in each year

**Year/myopia (D)**	**OK lens**^**#**^	**Atropine**^**$**^
1	0.29 ± 0.31	0.31 ± 0.19
2	0.27 ± 0.24	0.35 ± 0.25
3	0.28 ± 0.31	0.32 ± 0.23
**Year/astigmatism (D)**	**OK**^**#**^	**Atropine**^**$**^
1	±0.08 ± 0.11	±0.03 ± 0.02
2	±0.08 ± 0.42	±0.09 ± 0.12
3	±0.12 ± 0.35	±0.11 ± 0.16
**Year/axial length (mm)**	**OK lens**^**#**^	**Atropine**^**$**^
1	0.28 ± 0.08	0.38 ± 0.09
2	0.30 ± 0.09	0.37 ± 0.12
3	0.27 ± 0.10	0.36 ± 0.08

^#^OK: Orthokeratology.

^$^0.125% atropine.

The change in mean cornea endothelium cell count was not significantly different between the OK lens and atropine group (change per year, ±38 cell/mm^2^ and ±30 cell/mm^2^; *p =* 0.785). The UCVA of the OK lens group was 0.2 logMAR (20/30) to -0.1 logMAR (20/16), and BCVA of the atropine group was 0.1 logMAR (20/25) to -0.1 logMAR (20/16) at 2 and 4 pm, respectively.

**Figure 1:**
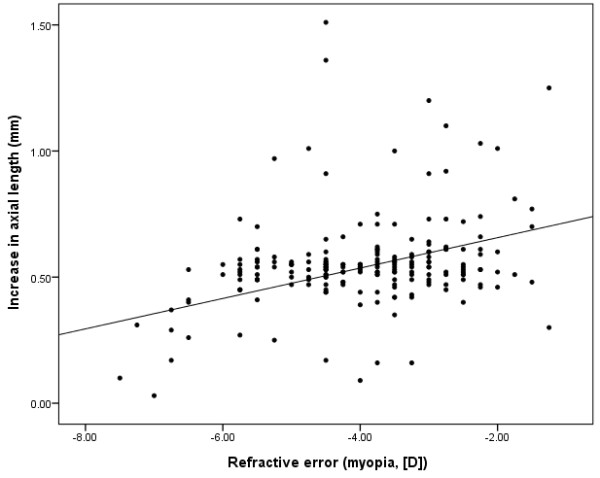
**Increases in axial length (mm) and refractive errors (myopia [D]) at baseline in the OK group.** A significant correlation was found between the increases in axial length and spherical equivalent refractive errors (myopia [D]) at the baseline. Pearson’s correlation coefficient: r = 0.259, *p* < 0.001.

**Figure 2:**
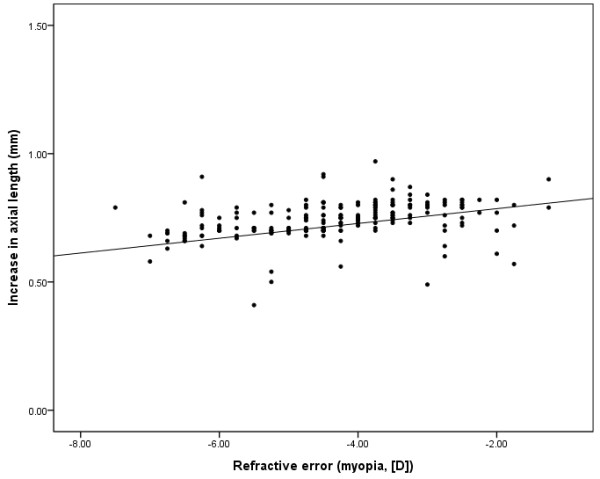
**Increases in axial length (mm) and refractive errors (myopia [D]) at baseline in the atropine used group.** A significant correlation was found between the increases in axial length and spherical equivalent refractive errors (myopia [D]) at the baseline. Pearson’s correlation coefficient: r = 0.169, *p* = 0.014.

To understand the relationship between the refractive error at baseline and increased axial length, Pearson’s correlation coefficient was employed. Significant correlation was found between these parameters in the OK lens group (Pearson’s correlation coefficient; *r* = 0.259, *p* < 0.001) as well as in the atropine group (*r* = 0.169, *p* = 0.014; Figures 1 and 2). The effect of decreasing the progression of axial length was more pronounced in high myopia patients than in low myopia patients for both groups. The regression coefficient (β) was higher in the OK lens group than in the atropine group (β = 0.060 [OK lens group] and 0.029 [atropine group], respectively).

In the OK lens group, the most common complication was allergic conjunctivitis; in 37 eyes (17.6%), there was an uncomfortable feeling such as itching during daytime, watery discharge on awaking, and requirement of drugs for relieving the symptoms. The care solution of OK lens used were “Boston Conditioning Solution and Boston Cleaner” (Bausch & Lomb Taiwan Ltd, Taiwan) or BIOCLEN Contact Lens Solution (BIOCLEN OPHTECS, Japan). Fifteen eyes (7.14%) showed superficial keratitis, which improved 3–7 days after terminating the use of OK lenses, without the need for drug administration and the OK lens were used continuously after re-education the taking care methods of the lens. No other complications, including corneal ulcers, were noted. In the atropine group, 2 eyes (1 patient [0.095%]) showed mild allergic blepharitis, which improved after topical application of anti-allergy medication. In OK lens group, the patients with myopia over then 5.75 D would use double reverse curves and dual geometric (DG) designs OK lens from Euclid Systems Corp. Their UCVA were better then 0.2 logMAR at 2–4 pm and just 2 patients needed spectacles at evening for taking lessons after school, none of the other needed spectacles in daily life. The major complaints of atropine application were photophobia during the day (35%), which could be resolved by photochromic lenses or sunglasses (72%), and poor near visual acuity (12%), which could be improved by multifocal lenses in most patients (96%). As for the effects of multifocal lenses and photochromic lenses for delaying myopia progression are also important subjects but are not the main themes in this study and need another investigation. No other evident abnormality was noted during the treatment period.

## Discussion and conclusions

Atropine is a well-known drug for treating myopia. In this study, OK lens is effective in slowing progression of myopia and increasing of axial length over a period of 3 years and is compatible with the effect of atropine. In our knowledge, this is the first paper compare the effect of atropine and Ok lens in controlling progression of myopia. In this study, we find that Ok lens is mild better then atropine in controlling axial length elongation and myopia progression. Besides, Ok lens users do not suffer photophobia and risk of inducing crowding of anterior chamber angle and increasing intraocular pressure (IOP) which may be induced by atropine. Moreover, the benefit of OK lens include that ceasing wearing glasses in daily life which bring convenience, better quality of life, no influence or near vision and somewhat good-looking. Nevertheless, there are still some drawbacks of OK lens, such as risk of cornea damage, infection and decreasing of cornea endothelium cell count; glare at night or vision decrease at evening are seen in OK lens users sometimes. There is no absolutely conclusion that OK lens or atropine is better, we just suggest that from the view of controlling myopia, Ok lens is useful methods.

The data presented in this study, the increase in axial length was 0.28 ± 0.08 mm per year in the OK lens group versus 0.37 ± 0.11 mm per year in the atropine group. In 2005, Cho et al. reported that axial length increased by 0.29 ± 0.27 mm in the OK lens group and 0.54 ± 0.27 mm in a control group treated with spectacles during a 2-year follow-up [30]. In 2009, Walline et al. reported similar findings; the mean increase in axial length after 2 years was 0.25 mm in the OK group and 0.57 mm in the control group [31]. In 2011, Kakita et al. obtained similar changes of 0.39 ± 0.27 mm in the OK lens group versus 0.61 ± 0.24 mm in the control spectacles group over 2 years [32]. The study of Walline et al. was performed with American patients, in whom the progression of myopia is reportedly slower than in the Asian population; this may explain the lesser increase in axial length in their study [31]. The results of Kakita et al. were obtained in Japan with an ethnic group similar to ours, and their results are similar to those of our study [32]. The increase in axial length in our OK lens group (0.28 ± 0.08 mm per year) was a little less than that reported by Cho et al. (0.29 ± 0.27 mm per year). The study of Cho et al. was performed in Hong Kong, in an area with high prevalence of myopia. Differences in the results may be because they used an ultrasonic A-mode device to measure axial length. In the present study, laser interferometry (IOLMaster; Carl Zeiss Meditec) was used to obtain noncontact measurements of axial length. This method has high reproducibility, and the non-contact procedure decreases the influences induced by compression of the cornea [33]. The increase in axial length in our atropine group was the smaller then the control groups of all the studies mentioned above. This can be understood by that 0.125% atropine is effective in decreasing myopia progression and axial length increase. Nevertheless, there were significant difference between the OK lens group and the 0.125% atropine group in quantity of axial length and myopic diopters. Moreover, there are data in our study worth pay attention, that is the standard deviation of atropine group in the second and third year is higher then in OK lens group, this might meant that the controlling myopia in this group is not very stable during the period.

The major limitation of our study is that the myopic diopter and axial length were checked every year after discontinuing use of OK lenses for only 3 weeks; this may not disturb the results of checking axial length but 3 weeks may not enough for the refractive error to completely recover to the baseline condition. However, patients in the OK lens group often relied on the OK lenses for their daily vision and could not discontinue use of the OK lens for 4–6 weeks [34], and 4–6 weeks has been proved to be sufficient for restoration of corneal curvature to the baseline condition so that the exact myopia diopter can be determined. This limitation is offset by the fact that the axial length, which is not influenced to a great extent by use of the OK lens, was also measured in this study, and a significant difference in axial length was found. Moreover, the included patients who completed the 3 years following-up without washout might be with satisfactory myopia control effect and lower or mild incidence of adverse effects; this induced the study biased towards successful cases. Nevertheless, the bias existed in both OK lens and atropine groups and cannot be neither avoided nor neglected.

Another limitation of our study is that age may influence the progression of myopia. A previous study demonstrated that myopia increased most remarkably at the age of 10–13 years; therefore, if we assessed only 10- to 13-year-old patients, the differences in myopic degree and axial length between the 2 groups were still observed, but to a lesser extent (*p =* 0.003 and *p =* 0.0035, respectively). However, the sample number was decreased after age stratification. Therefore, a larger study sample is required for future studies based on age stratification.

Allergic conjunctivitis happened in 37 eyes (17.6%) of OK lens. The care solution of Ok lens used were “Boston Conditioning Solution and Boston Cleaner” (Bausch & Lomb Taiwan Ltd, Taiwan) or BIOCLEN Contact Lens Solution (BIOCLEN OPHTECS, Japan). There was no evident difference that which solution will induce allergic conjunctivitis in our patients. The allergic conditions would subside after gave topic anti-histamine for 2 days without change care solutions. The allergic conjunctivitis might due to the warm and moist climate in our country. Patients selected the care solution at their own convenience. None of the patients had complains of the care solution.

Some of OK lens users exhibited UCVA greater than 0.2 logMAR (20/30) or had obvious fluctuations in diurnal UCVA. These patients often had a flatter cornea curvature, irregular cornea surface, or tight eyelids. The limitations of OK lenses are obvious, and the tight eyelids of Asian subjects are a substantial concern. Although the new multiple-zone, reverse-geometry lenses have a better outcome, not all patients are satisfied with the OK lens. No severe corneal infection occurred in this study group; it is important to educate the patients and their families about maintenance of healthy habits and appropriate handling of OK lenses.

Patients with high myopia at baseline showed less severe increase in axial length than those with low myopia in both the OK lens and atropine groups. The linear correlation was more significant in the OK lens group then in the atropine group (*r* = 0.259 versus *r* = 0.169). This phenomenon may occur because peripheral refraction changes are more evident in high myopia patients with OK lenses [32]. Myopic eyes usually have relative hyperopic defocus in the periphery, because the eye is elongated along the optic axis. Recent studies suggest that peripheral vision can influence axial length in human eyes, potentially altering the central refractive error and its development because of the emmetropization effect of eye growth. Conversion of relative peripheral hyperopia to relative peripheral myopia is a good method to limit the axial elongation that leads to myopia [26,35], and OK lenses appear to be an excellent option for achieving this objective. OK lenses appear to be a good tool to control high myopia. OK lenses create a small central zone and a smaller central visual field in high myopia patients comparing with lower myopia ones. At the same time, high myopia patients were with a greater area of the peripheral field remaining myopic. If changes of peripheral refraction are the primary reason for slowing progression of myopia, this would be expected to decrease axial length elongation especially in high myopia patients [36]. More human and animal studies are required to clearly test this hypothesis. However, clear central vision is essential for preventing defocus-inducing myopia.

The efficacy and safety of atropine is undoubted. Nevertheless, increasing intraocular pressure and photo-stress of the crystalline lens, retina, photophobia and poor near vision are often to be concerned in the patients using atropine. Low concentration atropine has proved useful in recent study that it can control myopia progression and decrease the side effects of high concentration atropine, low concentration atropine might an another good choice. OK lens with well care and hygiene may be one of the good policies to prevent progression of myopia and it does not just bring convenience for myopic patient to remove glasses in daytime. The combined use of OK lenses and atropine is a potential treatment for myopia progression and is being tested at our department. Hope this can give a new concept in delaying progression of myopia and can go a step further to resolve myopic problems.

## Abbreviations

IOL: Intraocular lenses; mAChR: Muscarinic acetylcholine receptor; OK: Orthokeratology; D: Diopters; logMAR: Logarithm of the minimum angle of resolution; UCVA: Un-corrected visual acuity; BCVA: Best corrected visual acuity.

## Competing interests

All authors declare that they have no competing interests.

## Authors’ contributions

HJL has collected the data of the trial, designed the study and drafted the manuscript. LW participated in the design of the study and critically revised the manuscript. FJT collected the data and participated in the design of the study. YYT participated in its design and coordination. LAC had collected the data of the trial and participated in its design and coordination. ALT had collected the data. YCH performed the statistical analysis has given final approval of the version to be published. All authors read and approved the final manuscript.

## Pre-publication history

The pre-publication history for this paper can be accessed here:

http://www.biomedcentral.com/1471-2415/14/40/prepub
